# Computational Study of the Coupled Mechanism of Thermophoretic Transportation and Mixed Convection Flow around the Surface of a Sphere

**DOI:** 10.3390/molecules25112694

**Published:** 2020-06-10

**Authors:** Amir Abbas, Muhammad Ashraf, Yu-Ming Chu, Saqib Zia, Ilyas Khan, Kottakkaran Sooppy Nisar

**Affiliations:** 1 Department of Mathematics, Faculty of Science, University of Sargodha, Sargodha 40100, Pakistan; amirabbas4693@gmail.com (A.A.); muhammad.ashraf@uos.edu.pk (M.A.); 2 Department of Mathematics, Huzhou University, Huzhou 313000, China; chuyuming@zjhu.edu.cn; 3 Hunan Provincial Key Laboratory of Mathematical Modeling and Analysis in Engineering, Changsha University of Science & Technology, Changsha 410114, China; 4 Department of Mathematics, COMSATS University Islamabad, Islamabad 40100, Pakistan; saqibzia81@hotmail.com; 5 Faculty of Mathematics and Statistics, Ton Duc Thang University, Ho Chi Minh City 72915, Vietnam; 6 Department of Mathematics, College of Arts and Sciences, Prince Sattam bin Abdulaziz University, Wadi Aldawaser 11991, Saudi Arabia; n.sooppy@psau.edu.sa

**Keywords:** thermophoretic, mixed convection, finite difference method, sphere, primitive variable formulation

## Abstract

The main goal of the current work was to study the coupled mechanism of thermophoretic transportation and mixed convection flow around the surface of the sphere. To analyze the characteristics of heat and fluid flow in the presence of thermophoretic transportation, a mathematical model in terms of non-linear coupled partial differential equations obeying the laws of conservation was formulated. Moreover, the mathematical model of the proposed phenomena was approximated by implementing the finite difference scheme and boundary value problem of fourth order code BVP4C built-in scheme. The novelty point of this paper is that the primitive variable formulation is introduced to transform the system of partial differential equations into a primitive form to make the line of the algorithm smooth. Secondly, the term thermophoretic transportation in the mass equation is introduced in the mass equation and thus the effect of thermophoretic transportation can be calculated at different positions of the sphere. Basically, in this study, some favorite positions around the sphere were located, where the velocity field, temperature distribution, mass concentration, skin friction, and rate of heat transfer can be calculated simultaneously without any separation in flow around the surface of the sphere.

## 1. Introduction

The present work deals with the analysis of the coupled mechanism of thermophoretic transportation and mixed convection around the surface of a sphere. In this study, a sphere is working as a heat source with a constant surface temperature, and the exact velocity components at the solid surface of a sphere are considered to be zero and approach an ambient condition far from the surface.

The sphere is a source of heat, which transmits its energy to its surroundings, where submicron particles are in motion and have low energy. These small particles receive energy from the sphere and gain momentum through the temperature gradient and this phenomenon is termed as thermophoretic motion. The force by which these particles are repelled far from the surface of a sphere is called thermophoretic force. Carl Ludwig was the first one to observe thermophoresis in liquid mixtures and in gas mixtures; it was reported by John Tyndall. The phenomenon of heat convection around the surface of a sphere takes place due to a temperature difference and thermophoretic motion. 

There are several applications of the thermophoresis phenomenon in industrial processes, like in the removal of minor-sized particles from streams of gas and identification of the channel of exhaust from the devices used in combustion processes. Its significance can be seen in particle deposition on the blades of turbines, the blackening of a gas glob of a kerosene lantern, and the filtration of impure gas. The disgusting of tools of gas turbines, corrosion occurring in heat exchangers, and clotting of condensing/evaporating fine mist is the contribution of thermophoretic deposition. In many of these applications, soot and combustion products deposit on cooled surfaces, which reduces the heat transfer efficiency by a considerable amount and may lead to pollution and fouling of the flow passages. 

The depositions of submicron particles drop the pressure and decrease the engine efficiency and enhance the fuel consumption. Some submicron particles like soot particles are acidic and can cause corrosion on surfaces. Experiments show that water droplets in turbines have a larger size range and are more than 90% of the vapor in the form of fog, which has a very small diameter. In cooled gas turbines, the blades are 300–400 K colder than the flow, and so thermophoresis phenomena are very significant in droplet deposition. Aircraft engines experience many difficulties when passing through suspended volcanic ash clouds as deposited particles can block the cooling holes of the turbines blades which are diverse applications of thermophoretic motion. Very similar problems can occur in heavily particle-driven flows, such as explosive eruption and sandstorms, which may damage airplane engines significantly. 

The thermophoresis mechanism has vast applications in the engineering field, such as a micro-scale thermophoresis instrument, which is specifically based on thermophoresis and measures the equilibrium binding events; it has been shown to have potential in drug discovery. It is used for the separation of different polymer particles in field flow fractionation. It is used in the manufacturing of optical fibers in vacuum deposition processes. Thermophoresis is used for the sampling of aerosol particles. Thermophoresis removes small gas particles from combustion devices and turbine blades to avoid accidents in nuclear reactors. It is used in electrostatic precipitators. It serves as a transport mechanism in fouling. Engineers use the thermophoresis phenomenon while they are designing air cleaning devices. Its uses are seen in chemical vapor deposition. Due to the variety of applications of mixed convection and thermophoretic motion, many researchers have presented this phenomenon in different flow geometries. 

The salient features of the material properties of fluid erupting from the boundary layer and in the plume region above the sphere were examined by Potter and Riley [1]. The analysis of thermophoretic force and natural convection flow by encompassing the particles’ deposition on a vertical surface was performed numerically in [2]. Riley [3] provided qualitative and reliable theoretical observations about convection flow around the heated sphere. He performed a numerical simulation of the governing equations for several finite several values of Prandtl as well as the Grashof number. Loyalka [4] investigated the effects of thermophoretic transportation on a single particle by solving the linearized Boltzmann equation numerically. Kang and Greif [5] found that there is a wide reduction in the deposition efficiency when there is no alignment of the torch with the target during the vapor deposition process.

A rigorous mathematical treatment of unsteady conjugate heat and the fluid flow mechanism about a solid sphere was achieved by Nguyen et al. [6], and the Chebyshev-Legendre spectral method has been implemented for the numerical results of the governing model. A technical note on the effects of thermophoresis for spheres made up of solid and liquid in air was presented by Li and Davis [7]. The influence of the surface mass through mixed convection over a vertical heated porous surface with a thermophoresis process was investigated by Selim et al. [8]. They computed the numerical results of the flow by employing the implicit finite difference and perturbation techniques. Chamkha and Pop [9] predicted the convective heat and fluid flow mechanism over the surface considered vertically fixed in porous medium and observed deposition effects on heat and mass transfer phenomenon. Chamkha et al. [10] encountered the influences of heat generation and absorption combined with thermophoresis on convective flow on a vertical flat plate.

The convective flow phenomenon through the sphere under the action of the generation of heat and variable viscosity were investigated numerically in [11,12]. Postelnicu [13] extended the work of Chamkha and Pop [9] by considering the thermophoretic deposition along a normal direction from the surface of the proposed geometry. He found that the general behavior of heat and fluid flow mechanisms in both studies is synchronized.

The analytical solutions of the Soret and Dufour phenomenon by considering the thermophoretic particle deposition with the inclusion of chemical reactions were simulated by Siva Raman et al. [14] by using a group theoretic method. They observed that the effects of thermophoresis are dominant as compared with thermal and mass diffusion. Musong and Feng [15] proposed the problem of heat and fluid flow about a heated sphere by incorporating the different ranges of the Richardson as well as the Reynolds’ number. They found that the relationship between the heat transfer rate and the Richardson number is very linear for wide ranges of arbitrary incident flow angles in the laminar flow regime. Malai et al. [16] concluded that the dependency factor of the thermophoretic force and velocity on the average temperature of the particle is nonlinear. Gompper et al. [17] elaborated the fascination, significance, and diverse physical aspects of the self-propelled particles’ motion. 

The viscous dissipation influences on mixed convection with periodic behavior of physical properties around the sphere were explored in [18] numerically. Burelbach et al. [19] measured the thermophoretic force on various polystyrene particles of a varying surface coating, negative charge, and size by using the bright field and came to the conclusion that colloids with the weakest zeta potential show the strongest thermophoretic effect. 

Theoretical investigations of the thermophoretic forces on nano-cylinders suspended in gas and a mixture of gases were performed by Wang et al. [20]. The comparison of the various forms of correction on the first-order derivative of the smooth particle hydrodynamics method is given in [21]. The authors’ main focus was to determine the one optimal kernel approximation. Chang and Keh [22] produced analytical results of the thermophoretic force induced by a spherical-shaped particle suspended in gas that has a uniform temperature gradient for a small Peclet number with the help of the asymptotic expansion method.

Yu et al. [23] devoted their efforts to focus on the phenomenon of thermophoresis produced by the nano-spherical-shaped convex particle. The gas considered by them had a non-uniform temperature filled in the region of the free molecule. The thermophoresis transportation of spherical particles suspended in gas within a spherical-shaped cavity along with its material properties were examined by Li and Keh [24] analytically. The characteristics of the shear stress and heat transfer rate around the surface of a sphere are discussed in [25].

Juan et al. [26] investigated the mixed convection heat transfer mechanism numerically and experimentally about a rectangular geometry whose sides were all adiabatic except the front, which was an isothermal side. The surface heating effects on a mixed convective fluid flow in a cylindrical geometry were examined by Osman et al. [27].

Niu and Palberg [28] proposed the study of the establishment and development of a modular approach regarding micro swimming. Xiong et al. [29] proposed the study of particle deposition caused by thermophoretic force by using a modified Markov model. Karim et al. [30] discussed the molten salt nanofluid as high-temperature fluid in a direct absorption solar collector system. They considered a two-dimensional computational fluid dynamics model of a direct absorption high-temperature molten salt nano-fluid concentrating solar receiver.

Minakov and Schick [31] presented analytical solutions of the heat transfer problem for local fast thermal perturbations around carbon nano tube CNT-polymer. Investigators have presented analytical and numerical studies of the phenomena of thermophoresis in different fluids considering different geometries in [32,33,34,35,36,37].

Khan et al. [38] performed a detailed analysis on the model and applications of nonofluid flow between infinite parallel plates suspended by micro cantilever sensors by adopting the effective Prandtl model. 

Gompper et al. [39] addressed the researchers of the present era about the different physical aspects and significance of the self-propulsion mechanism and motility of a huge number of identical agents by the help of the 2020 self-motile active matter roadmap.

Based on the above literature review, in the current study, a parametric analysis was conducted to analyze the effect of the dimensionless parameters involved in the flow model on the velocity distribution, temperature distribution, and mass concentration, as well as their derivatives along with the thermophoretic velocity. For this purpose, the finite difference method and a built-in numerical scheme boundary value problem of fourth order code BVP4C was used and the main numerical findings are displayed graphically and in a tabular form.

## 2. Mathematical Formulation

**Figure 1:**
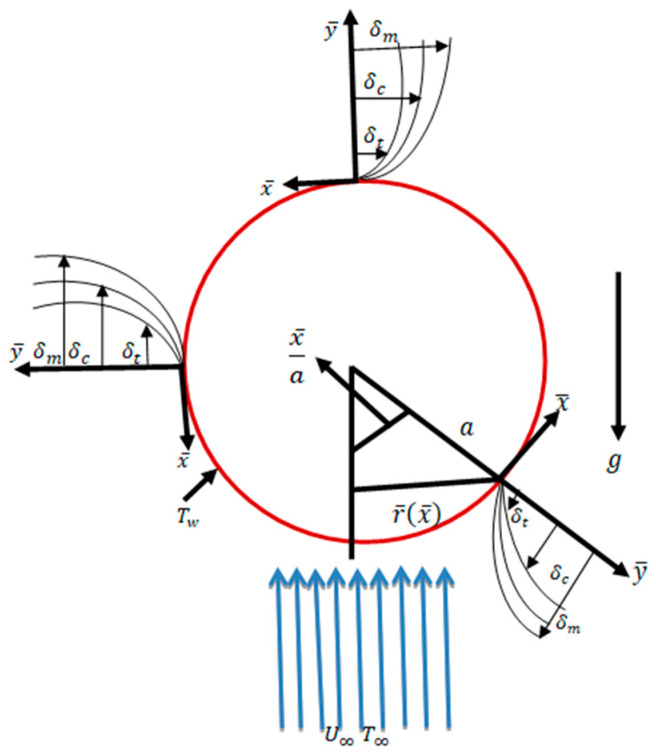
Interpretation of flow geometry.

In steady, incompressible, laminar, viscous, and two-dimensional mixed convection flows along a sphere surface with a radius *a*, the temperature and mass concentration on the surface of the sphere are *T_W_* and *C_W_,* respectively, with conditions $T_{w}>T_{\infty }$ and $C_{w}>C_{\infty }$, where $T_{\infty }$ and $C_{\infty }$ are the ambient temperature and mass concentration, respectively. The $\;\bar{x}$ and $\bar{y}$ coordinates are parallel and normal to the surface as shown in Figure 1. The corresponding velocity components in the $\bar{x}$ and $\bar{y}$ directions are $\bar{u}$ and $\bar{v}$, respectively. The dimensionless flow equations are given below:

(1) $$\frac{\;\partial \left(r\;u\right)}{\partial x}+\frac{\partial \left(r\;v\right)}{\partial y},$$

(2) $$u\frac{\partial u}{\partial x}+v\frac{\partial u}{\partial y}=\frac{\partial^{2}u}{\partial y^{2}}+\lambda_{t}\theta sinx+\lambda_{c}\phi sinx,$$

(3) $$u\frac{\partial \theta }{\partial x}+v\frac{\partial \theta }{\partial y}=\frac{1}{Pr}\frac{\partial^{2}\theta }{\partial y^{2}},$$

(4) $$u\frac{\partial \phi }{\partial x}+v\frac{\partial \phi }{\partial y}=\frac{1}{Sc}\frac{\partial^{2}\phi }{\partial y^{2}}-\frac{\partial \left(v_{t}\phi \right)}{\partial y}.$$

By following Potter [1], r= sinx, is the radial distance from the axis of the symmetric to the surface of the sphere. In Equations (1)–(4), u and v represent the velocity components along the x and y directions, respectively.

In Equation (2), the physical variable *θ* denotes the dimensionless temperature of the fluid flow domain. In Equation (3), the variable *φ* designates the dimensionless mass concentration within the domain of the flow mechanism. The symbols Pr $=\frac{\nu }{\alpha }$,$\;\lambda_{t}=\frac{Gr_{t}}{Re^{2}}$, Sc $=\frac{\nu }{D_{m}}$, and $\;\lambda_{c}=\frac{Gr_{c}}{Re^{2}}$ are the Prandtl number, mixed convection parameter, Schmidt number, and modified mixed convection parameter, respectively. Here, $Gr_{t}=\frac{g\beta_{t}\left(T-T_{\infty }\right)a^{3}}{\nu^{2}}$,$\;Re=\frac{U_{\infty }a}{\nu }$, and $Gr_{c}=\frac{g\beta_{c}\left(C-C_{\infty }\right)a^{3}}{\nu^{2}}$ are theGrashof number, Reynolds number, and modified Grashof number, respectively. The variable *ν_t_* in Equation (4) is called the thermophoretic velocity and is defined below:

$$v_{t}=-\frac{k}{\theta +N_{t}}\frac{\partial \theta }{\partial y},$$

where k is the thermophoretic coefficient and $N_{t}=\frac{\Delta T}{T_{\infty }}$ is the thermophoresis parameter.

The related boundary conditions for the modeled problems are:

u=0,v=0,  θ=1,  φ=1at y=0,

(5) u→1,  θ→0,  φ→0, as y→∞.

## 3. Solution Methodology

The dimensionless governing Equations (1)–(4) associated with the boundary conditions (Equation (5)) were converted to a form that can be integrated conveniently by utilizing the primitive variable formulation (PVF),which is employed in [18] and [25]. We used the following set of primitive variables:

$$u\left(x,y\right)=U\left(X,Y\right),\;\;Y=x^{-\frac{1}{2}}y,\;\;\;v_{t}\left(x,y\right)=x^{-\frac{1}{2}}V_{t}\left(X,Y\right),$$

(6) $$\theta \left(x,y\right)=X^{-1}\theta \left(X,Y\right),\;\;v=x^{-\frac{1}{2}}V\left(X,Y\right),\;X=x,\;\varphi \left(x,y\right)=X^{-1}\varphi \left(X,Y\right).\;$$

The obtained transformed Equations (1)–(4) are:

(7) $$XU\;cosX+\left(X\frac{\partial U}{\partial X}-\frac{Y}{2}\frac{\partial U}{\partial Y}+\frac{\partial V}{\partial Y}\right)\;sinX=0,$$

(8) $$XU\frac{\partial U}{\partial X}+\left(V-\frac{YU}{2}\right)\frac{\partial U}{\partial Y}=\frac{\partial^{2}U}{\partial Y^{2}}+\lambda_{t}\theta \;\sin X+\lambda_{c}\varphi \;\sin X,$$

(9) $$XU\frac{\partial \theta }{\partial X}+\left(V-\frac{YU}{2}\right)\frac{\partial \theta }{\partial Y}-U\theta =\frac{1}{Pr}\frac{\partial^{2}\theta }{\partial Y^{2}},\;$$

(10) $$XU\frac{\partial \varphi }{\partial X}+\left(V-\frac{YU}{2}\right)\frac{\partial \varphi }{\partial Y}-U\varphi =\frac{1}{Sc}\frac{\partial^{2}\varphi }{\partial Y^{2}}-\frac{\partial \left(V_{t}\varphi \right)}{\partial Y},\;$$

where $V_{t}=-\frac{k\;}{\theta +{X\;N}_{t}}\frac{\partial \theta }{\partial Y}$.

Correspondingly, the transformed boundary conditions are:

U=0, V=0, θ=1, φ=1,at  Y=0,

(11) U→1,θ →0, φ→0  as Y→∞.

### 3.1. Solution Technique

The solution of the transformed system of Equations (7)–(10) with boundary conditions (Equation (11)) were obtained by using the finite difference method. Along the *x*-axis, the backward difference and along the *y*-axis, the central difference was applied. The solution procedure is explained below:

(12) $$\frac{\;\partial U}{\partial X}=\frac{U_{\left(i,j\right)}-U_{\left(i,j-1\right)}}{\Delta X},$$

(13) $$\frac{\partial U}{\partial Y}=\frac{U_{\left(i+1,j\right)}-U_{\left(i,-1,j\right)}}{2\Delta Y},\;$$

(14) $$\frac{\;\partial^{2}U}{\partial Y^{2}}=\frac{U_{\left(i+1,j\right)}-2U_{\left(i,j\right)}+U_{\left(i,-1,j\right)}}{\Delta Y^{2}}.\;$$

By using Equations (12)–(14) in Equations (7)–(10) along with the boundary conditions given in Equation (11), the following system of algebraic equations was obtained:

Continuity equation:

(15) $$\;V_{\left(i+1,j\right)}=V_{\left(i-1,j\right)}-2\frac{\Delta Y}{\Delta X}X_{i}\left(U_{\left(i,j\right)}-U_{\left(i,j-1\right)}\right)+\frac{Y_{j}}{2}\left(U_{\left(i+1,j\right)}-U_{\left(i-1,j\right)}\right)-2\Delta YX_{i}\frac{cosX_{i}}{\sin X_{i}}U_{\left(i,j\right)}.$$

Momentum equation:

(16) $$\begin{array}{l} \left(1+\frac{\Delta Y}{2}(V_{\left(i,j\right)}-\frac{Y_{j}}{2}U_{\left(i,j\right)}\right)U_{\left(i-1,j\right)}+\left(-2-\frac{\Delta Y^{2}}{\Delta X}X_{i}U_{\left(i,j\right)}\right)U_{\left(i,j\right)}+\left(1-\frac{\Delta Y}{2}\left(V_{\left(i,j\right)}-\frac{Y_{j}}{2}U_{\left(i,j\right)}\right)\right)U_{\left(i+1,j\right)}=\\ -\Delta Y^{2}sinX_{i}\;(\lambda_{t}\theta_{\left(i,j\right)}+\lambda_{c}\varphi_{\left(i,j\right)})-\;\frac{\Delta Y^{2}}{\Delta X}X_{i}U_{\left(i,j\right)}U_{\left(i,j-1\right)}. \end{array}$$

Energy equation:

(17) $$\begin{array}{l} \left(\frac{1}{Pr}+\frac{\Delta Y}{2}\left(V_{\left(i,j\right)}-\frac{Y_{j}}{2}U_{\left(i,j\right)}\right)\right)\theta_{\left(i-1,j\right)}+\left(-\frac{2}{Pr}+\Delta Y^{2}U_{\left(i,j\right)}\left(1-\frac{X_{i}}{\Delta X}\right)\right)\theta_{\left(i,j\right)}+\;\left(\frac{1}{Pr}-\frac{\Delta Y}{2}\left(V_{\left(i,j\right)}-\frac{Y_{j}}{2}U_{\left(i,j\right)}\right)\right)\theta_{\left(i+1,j\right)}=\\ -\frac{\Delta Y^{2}}{\Delta X}X_{i}U_{\left(i,j\right)}\theta_{\left(i,j-1\right)}. \end{array}$$

Mass concentration equation:

(18) $$\begin{array}{l} \left(\frac{1}{Sc}+\frac{\Delta Y}{2}\left(V_{t}{}_{\left(i,j\right)}+V_{\left(i,j\right)}-\frac{Y_{j}}{2}U_{\left(i,j\right)}\right)\right)\varphi_{\left(i-1,j\right)}+\left(-\frac{2}{Sc}+\Delta Y^{2}U_{\left(i,j\right)}\left(1-\frac{X_{i}}{\Delta X}\right)\right)\varphi_{\left(i,j\right)}+(\frac{1}{Sc}-\frac{\Delta Y}{2}(V_{t}{}_{\left(i,j\right)}+\\ V_{\left(i,j\right)}-\frac{Y_{j}}{2}U_{\left(i,j\right)}))\varphi_{\left(i+1,j\right)}=-\frac{\Delta Y^{2}}{\Delta X}X_{i}U_{\left(i,j\right)}\varphi_{\left(i,j-1\right)}, \end{array}$$

where the discretized thermophoretic velocity is:

$$V_{t}{}_{\left(i,j\right)}=-\frac{k}{\left(\theta_{\left(i,j\right)}+X_{i}N_{t}\right)}\frac{\theta_{\left(i+1,\;j\right)}-\theta_{\left(i-1,j\right)}}{2\Delta Y}.$$

The corresponding boundary conditions are:

$$U_{\left(i,j\right)}=0,\;\;\;\;\;V_{\left(i,j\right)}=0,\;\;\;\;\;\theta_{\left(i,j\right)}=1,{\;\varphi }_{\left(i,j\right)}=1,\;\mathrm{as}\;Y_{j}=0,$$

(19) $$U_{\left(i,j\right)}\to 1,\;\;\;\;\theta_{\left(i,j\right)}\;\to 0,\;\;{\;\varphi }_{\left(i,j\right)}\to 0,\;\;\;\;Y_{j}\to \infty .$$

Here, in *x* the and *y* directions, variables *U* and *V*, are components of the velocity, respectively. The temperature and mass distributions are θ and φ, respectively, where *i*, *j*, are mesh points along the *x* and *y* directions. Moreover, the Gaussian elimination technique was used to solve the set of algebraic equations given in Equations (16)–(18) along with the boundary conditions (Equation (19)). The values of the variables *U*, *θ*, and *φ* and their derivatives, along with *V_t_*, were calculated at each grid point with the help of the computing tool FORTRAN and the computed results were displayed graphically with the utilization of Tecplot 360. Additionally, the results are displayed in tabular form for different parametric conditions. At each position, the iteration process continues until the convergence criterion for all the variables 10^−5^ is achieved and satisfies the boundary condition at the surface and asymptotically far from the surface. The convergence criteria for *U*, *θ*, and *φ* at each step for the 20 by 20 mesh size is given by:

(20) $$max\left|U^{n+1}-U^{n}\right|\leq 10^{-5},\;max\left|\theta^{n+1}-\theta^{n}\right|\leq 10^{-5}\;\mathrm{and}\;max\left|\varphi^{n+1}-\varphi^{n}\right|\leq 10^{-5}.\;$$

In keeping with the view of the convergence criteria given in Equation (20) and by seeing the boundary conditions given in Equation (19), it can been seen that in each graph, the obtained results within the given boundary conditions are satisfied at the surface and very strong asymptotic behavior is observed far from the surface within the defined mesh size. It is important to mention that the CPU run time was 32 s to run one complete loop based on two values of one parameter. 

### 3.2. Group of Stream Function Formulation

Now, attention is paid to determine the similar solutions of Equations (1)–(4) associated with the boundary conditions (Equation (5)). For this purpose, the non-dimensional form of the partial differential Equations (1)–(4) was transformed along with the boundary conditions of Equation (5) into a system of ordinary differential equations by using an appropriate group of stream function formulations. By following [9], the following similarity variable formulation was introduced as:

(21) $$X=x,\;\eta =\frac{y}{X^{1/2}},\;\psi \left(x,y\right)=X^{\frac{1}{2}}f\left(\eta \right),\;\theta \left(x,y\right)=\theta \left(\eta \right),\varphi \left(x,y\right)=\varphi \left(\eta \right),\;\;\;$$

where η is the similarity variable, ψ is the non-dimensional stream function usually taken as ru=∂(rψ)/∂y, and rv=−∂(rψ)/∂x, θ(η) is the non-dimensional temperature profile and φ(*η*) is the non-dimensional concentration profile. Using Equation (21), the equation of continuity (Equation (1)) is satisfied automatically, whereas Equations (2)–(4) reduce to the following similarity equations:

(22) $$f^{\prime \prime \prime }+\left(\frac{1}{2}+XcotX\right)ff^{\prime \prime }+XsinX\left(\lambda_{t}\theta +\lambda_{c}\varphi \right)=0,\;$$

(23) $$\frac{1}{Pr}\theta \prime \prime +\left(\frac{1}{2}+XcotX\right)f\theta {}^{\prime }=0,\;$$

(24) $$\frac{1}{Sc}\varphi^{\prime \prime }+\left(\frac{1}{2}+XcotX\right)f\varphi^{\prime }+\frac{k}{Nt+\theta }\left(\theta^{\prime }\varphi^{\prime }+\varphi \theta^{\prime \prime }-\frac{\varphi \theta^{\prime 2}}{Nt+\theta }\right)=0.\;$$

The associated boundary conditions are:

$$f=0,f^{\prime }=0,\;\;\theta =1,\varphi =1\;\mathrm{at}\;\eta =0,$$

(25) $$f^{\prime }\to 1,\;\;\theta \to 0,\;\;\varphi \to 0,\;\;\mathrm{as}\;\eta \to \infty .$$

The transformed thermophoretic velocity is given by:

$$\;V_{t}=-\frac{k\;}{\theta +{\;N}_{t}}\theta^{\prime }\left(0\right).$$

The transformed system of ordinary differential equations of Equations (22)–(24) with the boundary conditions of Equation (25) were solved by using the built-in numerical solver BVP4C. Here, BVP4C stands for the boundary value problem fourth order. The comparison of the solution of the flow model for some physical quantities obtained by finite difference method FDM and boundary value problem of fourth order code BVP4C is given in Table 1, Table 2, Table 3, Table 4, Table 5 and Table 6, and it is concluded that the results obtained by both schemes are in good agreement, which shows the validation of the solutions obtained by FDM applied to the primitive form of the partial differential equations.

## 4. Results and Discussion

In the current analysis, simulation was performed for different values of the controlling parameters, including the Prandtl number Pr, mixed convection parameter *λ_t_*, Schmidt number Sc, thermophoresis parameter *N_t_*, thermophoretic coefficient κ, and modified mixed convection parameter *λ_C_* on the velocity field *U*, temperature field θ, concentration distribution φ, and thermophoretic velocity *V_t_* around different positions of the surface of the sphere. Additionally, under the effect of these parameters, the numerical solutions of skin friction, the rate of heat transfer, and the rate of mass transfer are displayed in graphical as well tabular forms. The details of the obtained numerical solutions along with the physical behavior are given in the subsequent paragraphs. Additionally, the numerical solutions of the governing problem obtained by FDM were validated by the MATLAB built-in numerical solver BVP4C. Comparison of the numerical solutions obtained by both methods is given in Table 1, Table 2, Table 3, Table 4, Table 5 and Table 6 for different values of the different dimensionless parameters.

**Figure 2:**
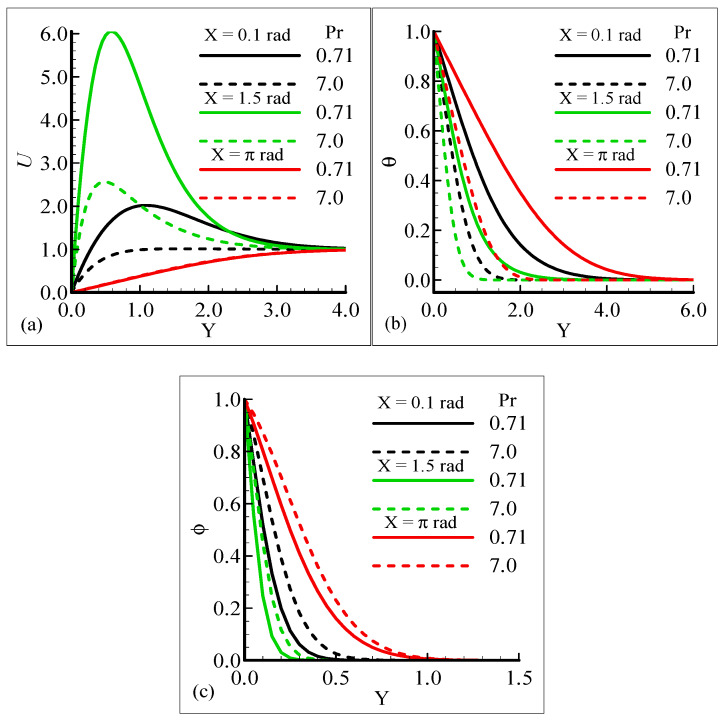
Graphical interpretation of variables (**a**) *U*, (**b**) *θ*, and (**c**) φ for several values of Pr when mixed convection parameter *λ_t_* = 50.0, modified mixed convection parameter *λ_C_* = 10.0, Schmidt number S_C_ = 1.0, thermophoretic coefficient κ = 1.0, and thermophoresis parameter *N_t_* = 10.0.

Figure 2a–c display the characteristics of the fluid velocity, the temperature of the domain, and the mass concentration for various values of the Prandtl number Pr around the surface of a sphere. From given plots, it is deduced that the velocity of the fluid flow is maximum at position *X* = 1.5 radian. We can infer that the temperature and mass concentrations are well behaved at position *X* = π radian for Pr = 0.71 and 7.0, respectively. 

Figure 2a–c, show that the phenomena of fluid velocity, temperature distribution, and mass concentration are vice versa; that is, the fluid velocity and temperature distribution are reduced but the opposite attitude for the case of the mass concentration is noted. It was expected because an enhancement in Pr leads to a rise in the viscous force and reduces the thermal diffusion, which causes the fluid velocity and temperature of the domain to decrease. Physically, it is true when Pr increases, the momentum diffusion is actually increased, and thermal diffusion is decreased. The momentum and thermal diffusions act like retarding forces, which causes the above said phenomenon in Figure 2a–c. 

**Figure 3:**
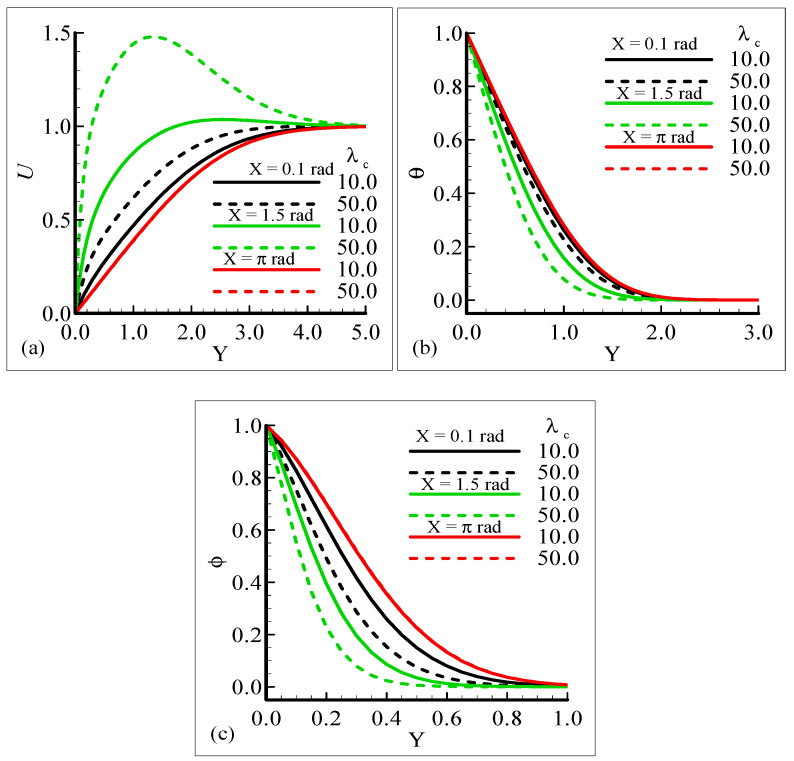
Graphical interpretation of the variables (**a**) *U*, (**b**) *θ*, and (**c**) *φ* for several values of *λ_C_* when the Schmidt number *Sc* = 10.0, mixed convection parameter *λ_t_* = 50.0, Prandtl number *Pr* = 7.0, thermophoretic coefficient κ = 1.0, and thermophoresis parameter *N_t_* = 10.0.

The impact of the various values of the modified mixed convection parameter, *λ_C_*, on the concentration, velocity, and temperature distributions is given through the results illustrated in Figure 3a–c. The considered range for the modified mixed convection parameter is *λ_C_* = 10.0 and *λ_C_* = 50.0, and very much spans over a wide range of possible operating conditions, which were observed. 

The figures show that the velocity profile is prominent at position *X* = 1.5 radian and has maximum motion for *λ_C_* = 50.0 radian. It is also noticed that the temperature and mass distributions have a maximum magnitude at position *X* = π radian and minimum magnitude at position *X* = 1.5 radian.

**Figure 4:**
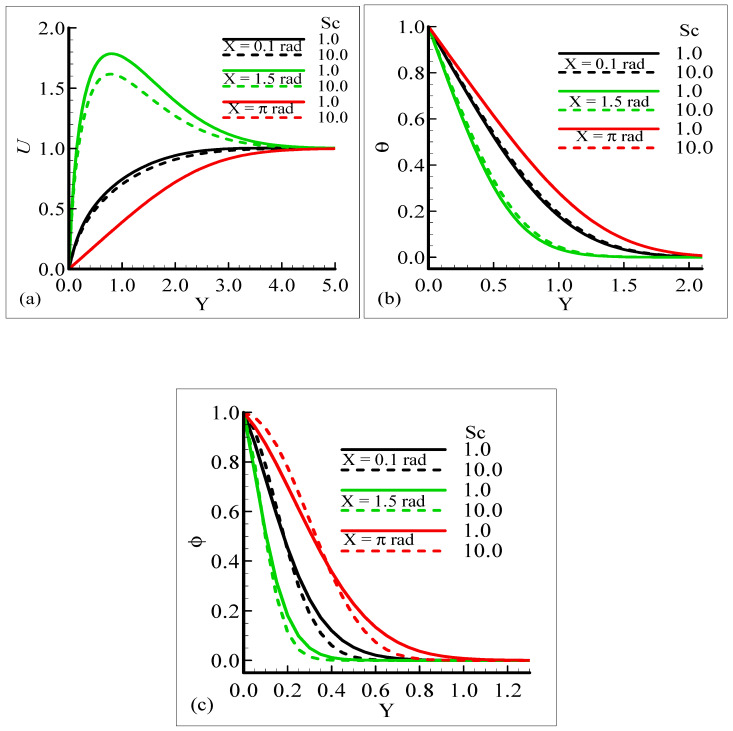
Graphical interpretation of variables (**a**) *U*, (**b**) *θ*, and (**c**) *φ* for several values of Sc when the mixed convection parameter *λ_t_* = 50.0, modified mixed convection parameter *λ_C_* = 10.0, Prandtl number *Pr* = 7.0, thermophoretic coefficient κ = 1.0, and thermophoresis parameter *N_t_* = 10.0.

Mathematically speaking, it can be noticed that the boundary condition in the figures is highly satisfied at the surface and far from the surface asymptotically. It is concluded that with an increase of *λ_C_*, the mass concentration difference is increased so the velocity of the fluid is enhanced and, consequently, the temperature and mass concentration decrease. Figure 4a–b present the typical velocity, temperature, and mass concentration profiles for three different positions around a sphere for Sc = 1.0 and 10.0, respectively. 

From the graphical results, it is predicted that an augmentation in Sc implies a significant decrease in the velocity and mass profiles and an increase in the temperature profile is observed. By viewing the behavior of the velocity, temperature, and mass profiles at several circumferential points around a sphere, it can be concluded that the velocity distribution attains a higher magnitude at *X* = 1.5 radian but the temperature and mass profiles achieve their higher magnitude at *X* = π radian. One noticeable point is that no difference is found in the velocity profile at *X* = π radian. The above said phenomenon takes place because with the increase of the Schmidt number, molecular diffusion is reduced, and viscous force is increased. It is predicted that with the increase of Sc, the viscosity of the fluid flow domain dominates the mass diffusion per unit volume, which reduces the fluid velocity at each position of the sphere. 

**Figure 5:**
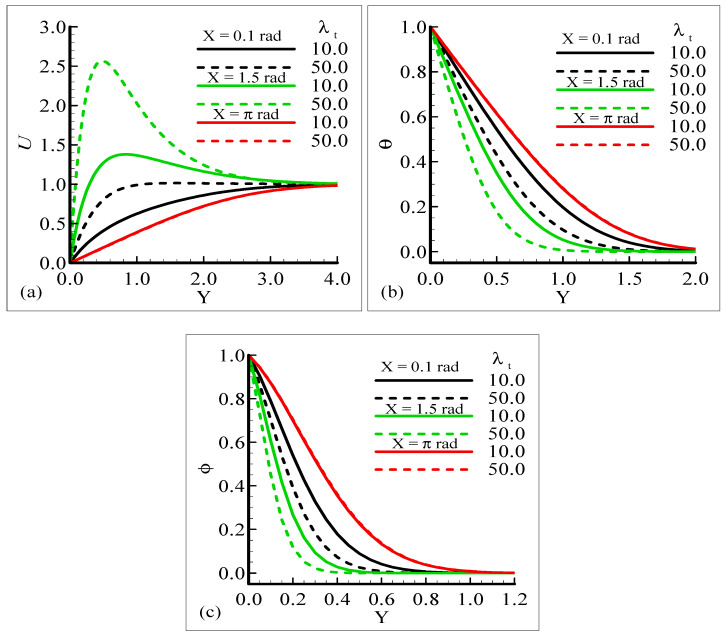
Graphical interpretation of the variables (**a**) *U*, (**b**) *θ*, and (**c**) *φ* for several values of *λ_t_* when the Schmidt number *Sc* = 1.0, modified mixed convection parameter *λ_C_* = 10.0, Prandtl number *Pr* = 7.0, thermophoretic coefficient κ = 1.0, and thermophoresis parameter *N_t_* = 10.0.

Figure 5a,b displays the effect of the mixed convection parameter, *λ_t_*, at different circumferential stations around the sphere on velocity, temperature, and mass profiles, respectively. The plots indicate that by increasing the values of the mixed convection parameter, *λ_t_*, from 10.0 to 50.0 by keeping the other parameters fixed, the velocity profile increases but the temperature and mass profiles decrease. It can be observed that the fluid velocity approaches its highest value at *X* = 1.5 radian, but the temperature and mass profiles approach maximum values at *X* = π radian. 

The results provide information indicating that no difference in the temperature profile and mass concentration is observed at position *X* = π radian corresponding to increasing values of *λ_t_*. In this mechanism, we noticed that the buoyancy force is working as a controlling parameter and is responsible for delaying or speeding up the fluid’s motion.

**Figure 6:**
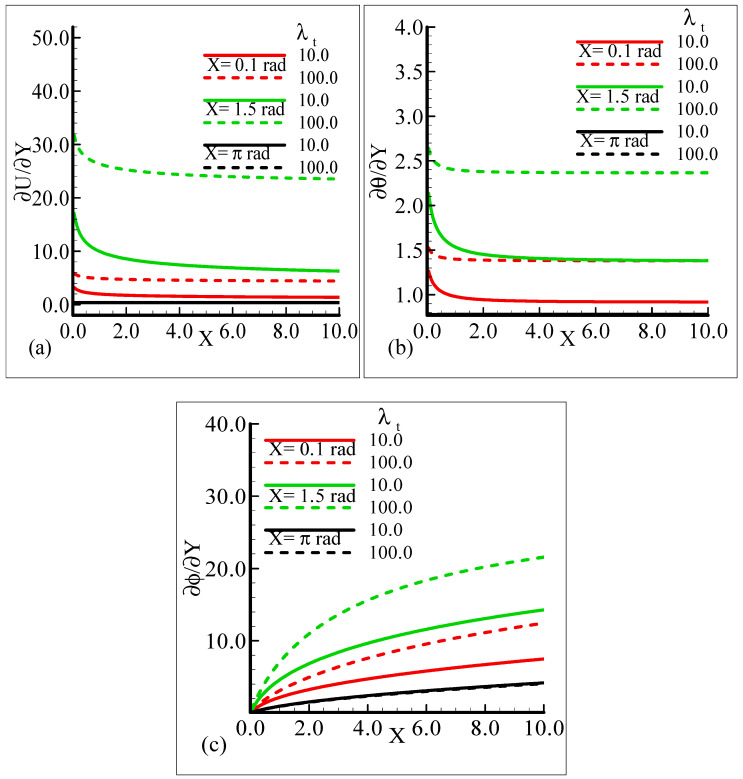
Graphical interpretation of the (**a**) skin friction, (**b**) rate of heat transfer, and (**c**) rate of mass transfer for several values of *λ_t_* when the Schmidt number *Sc* = 10.0, modified mixed convection parameter *λ_C_* = 50.0, Prandtl number *Pr* = 7.0, thermophoretic coefficient κ = 1.0, and thermophoresis parameter *N_t_* = 10.0.

In Figure 6a–c, the behavior of the skin friction, heat transfer rate, and mass transfer rate for several values of the mixed convection parameter, *λ_t_* = 10.0, 100.0, when the other parameters remain fixed is highlighted. We can see that as *λ_t_* is enhanced, the slopes of the velocity, temperature, and mass concentration profiles increase at positions *X* = 0.1 radian and *X* = 1.5 radian around a sphere, but at position *X* = π radian, all of the three physical quantities show no reasonable change corresponding to an increase in *λ_t_*.

**Figure 7:**
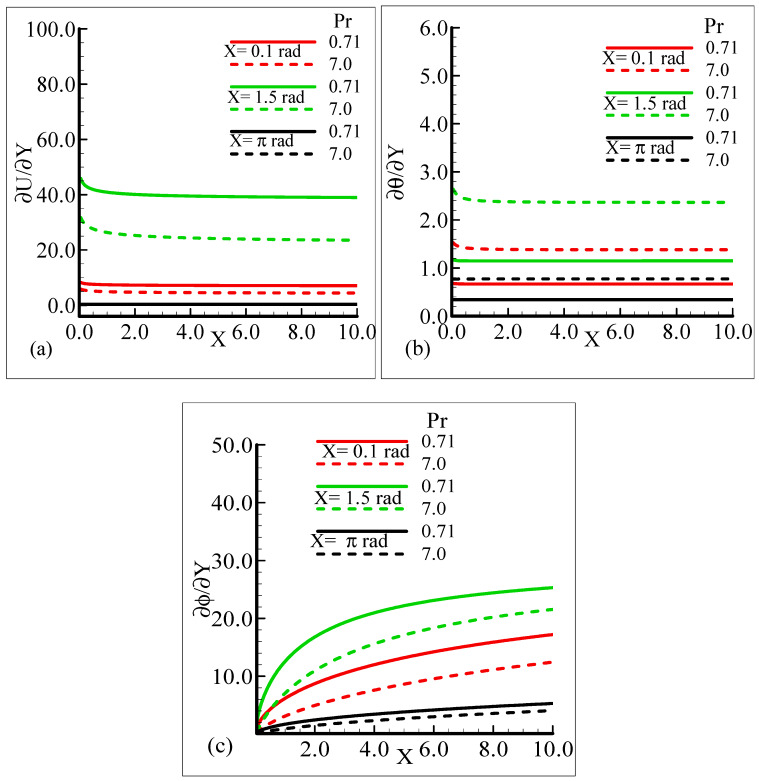
Graphical interpretation of the (**a**) skin friction, (**b**) rate of heat transfer, and (**c**) rate of mass transfer for several values of Pr when the Schmidt number *Sc* = 10.0, modified mixed convection parameter *λ_C_* = 50.0, mixed convection parameter, *λ_t_* = 100.0, thermophoretic coefficient κ = 1.0, and thermophoresis parameter *N_t_* = 1.0.

It can be observed that all three quantities are magnified at position *X* = 1.5 radian, and lower magnifications are noted at *X* = π radian. Figure 7a–b depict that for the Prandtl number Pr = 0.71 and 7.0, by keeping the other parameters fixed, skin friction decreases at *X* = 0.1 radian and *X* = 1.5 radian, but no change is observed at *X* = π radian. 

**Figure 8:**
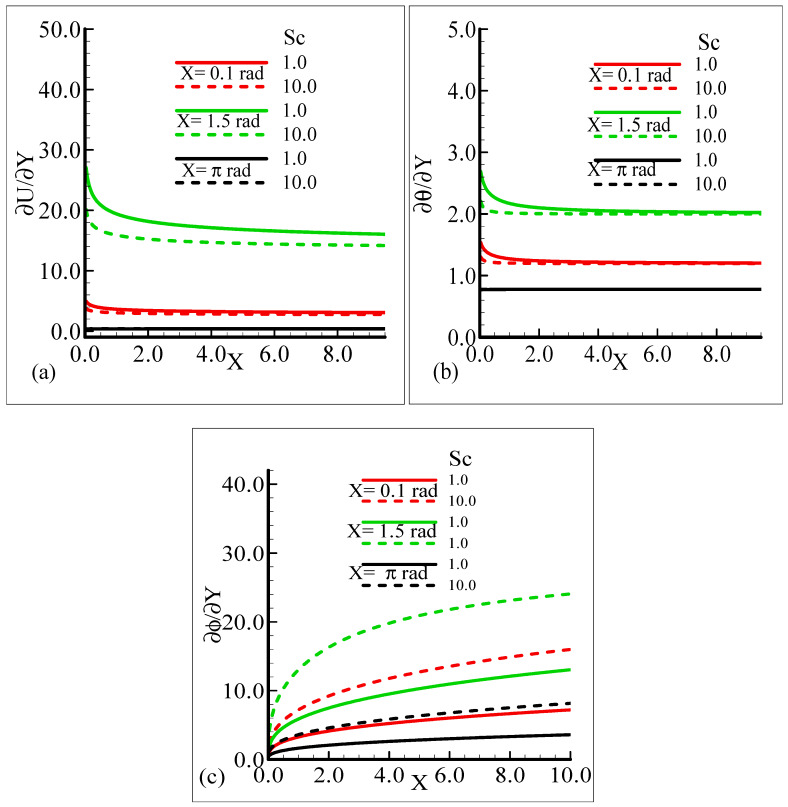
Graphical interpretation of the (**a**) skin friction, (**b**) rate of heat transfer, and (**c**) rate of mass transfer for several values of Sc when the mixed convection parameter, *λ_t_* = 50.0, modified mixed convection parameter *λ_C_* = 50.0, Prandtl number, Pr = 7.0, thermophoretic coefficient κ = 1.0, and thermophoresis parameter *N_t_* = 0.5.

On the other hand, the heat transfer rate and the mass transfer rate increase and decrease, respectively, with the augmentation in Pr. It can be noted that all three quantities secure their highest values at position *X* = 1.5 radian around a sphere. Figure 8a,b reveal the typical variations in skin friction, and the heat transfer rate along with the mass transfer rate at the expense of distinct Schmidt number values when the rest of the physical parameters are kept constant. 

**Figure 9:**
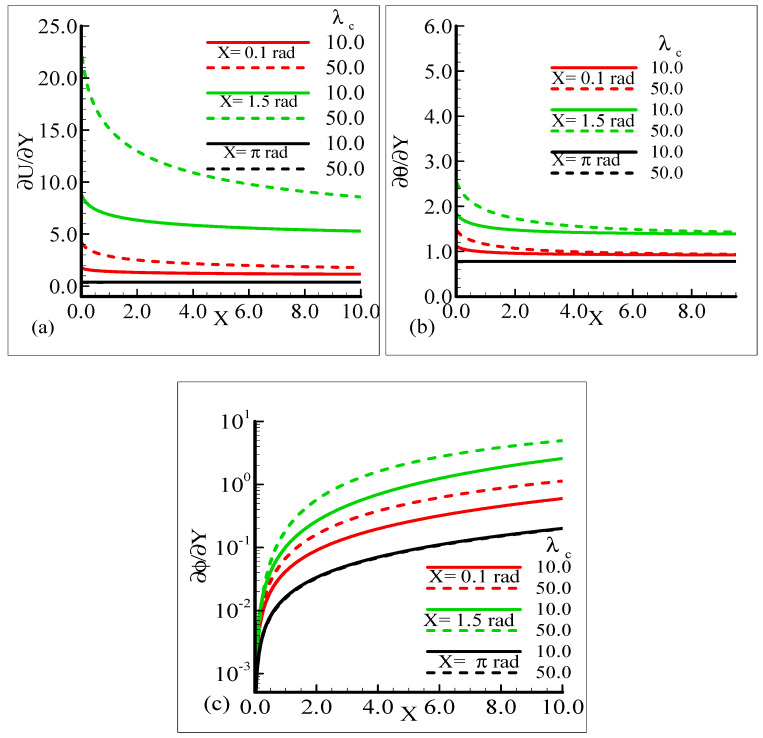
Graphical interpretation of the (**a**) skin friction, (**b**) rate of heat transfer, and (**c**) rate of mass transfer for several values of *λ_C_* = 10.0, 50.0 when the Schmidt number Sc = 10.0, mixed convection parameter *λ_t_* = 10.0, Prandtl number Pr = 7.0, thermophoretic coefficient κ = 1.0, and thermophoresis parameter *N_t_* = 10.0.

As Sc is increased, it gives rise to a loss of skin friction and the rate of heat transfer, and an increase in the rate of mass transfer is observed. It is shown that skin friction at position *X* = 1.5 radian achieves its maximum value and minimum value at *X* = π radian, and similar results are observed for the heat and mass transfer rates. Figure 9a–c elaborate on the effect of various estimations of *λ_C_* on the skin friction, heat transfer rate, and mass transfer rate. 

From the graphical demonstration, it is shown that the effect of modified mixed convection on skin friction along with the heat and mass transfer rates is more profound at position *X* = 1.5 radian. Further, in all cases, the amount of skin friction with the heat and mass transfer rate is dominated at position *X* = π radian.

**Figure 10:**
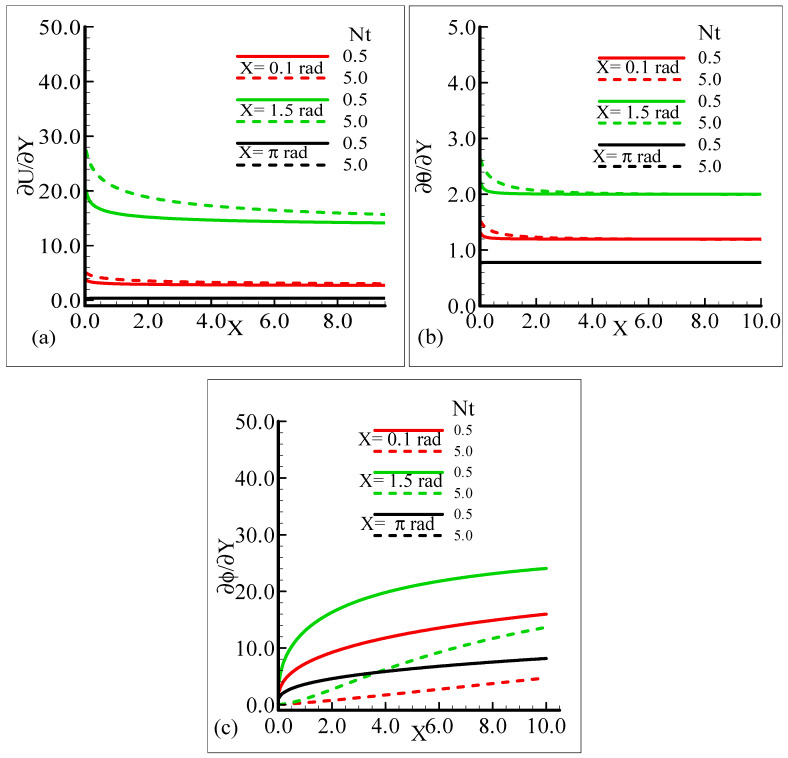
Graphical interpretation of the (**a**) skin friction, (**b**) rate of heat transfer, and (**c**) rate of mass transfer for several values of *N_t_* = 0.5, 5.0 when the Schmidt number Sc = 10.0, modified mixed convection parameter *λ_C_* = 50.0, Prandtl number Pr = 7.0, thermophoretic coefficient κ = 1.0, and thermophoresis parameter *λ_t_* = 50.0.

The behavior of the skin friction, rate of heat, and mass transfer under the influence of different values of the thermophoresis parameter, *N_t_*, when other parameters involved in the flow model are kept fixed is highlighted in Figure 10a–c. It can be viewed that around different positions of a sphere, when the thermophoresis parameter increases, the skin friction and rate of heat transfer are enhanced but the mass transfer rate decreases. 

By observing the variations in the skin friction and the heat and mass transfer rate around three different positions of a sphere, it is revealed that all of the aforementioned thermo physical quantities attain the highest values at *X* = 1.5 radian.

**Figure 11:**
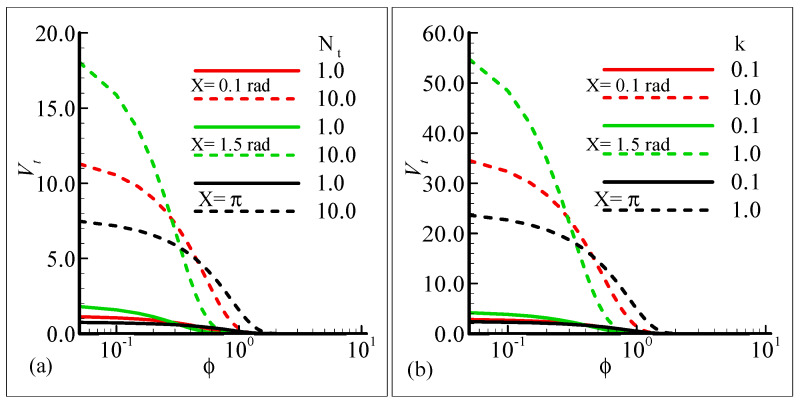
Graphical interpretation of the thermophoretic velocity (**a**) against φ for several values of *N_t_* = 1.0, 10.0 when the Prandtl number Pr = 7.0, thermophoretic coefficient κ = 1.0, Schmidt number Sc = 10.0, mixed convection parameter *λ_t_* = 50.0, and modified mixed convection parameter *λ_C_* = 10.0; and (**b**) against φ for several values of the thermophoretic coefficient κ = 0.1, 1.0 when the Prandtl number Pr = 7.0, Schmidt number Sc = 10.0, mixed convection parameter *λ_t_* = 10.0, modified mixed convection parameter *λ_C_* = 50.0, and thermophoresis parameter *N_t_* = 10.0.

Figure 11a,b depict the influence of the thermophoresis parameter, *N_t_*, and thermophoretic coefficient, κ, on the thermophoretic velocity, *V_t_*, at three different stations around the surface of a sphere against the volume fraction of the submicron particles. In these figures, it is shown that the thermophoretic velocity at the surface of the sphere is higher at position *X* = 1.5 radian, and in both cases, it approaches zero as the fraction or particle size is increased. Further, the curves in the graph intersect at a horizontal axis due to the mixing attitude of the thermophoresis particles.

Table 1, Table 2, Table 3, Table 4, Table 5 and Table 6 show a comparison of the results for the skin friction, rate of heat transfer, and rate of mass transfer for different values of the mixed convection parameter, *λ_t_*, and thermophoretic coefficient, κ, when the rest of the physical parameters are kept constant at a favorable point *X* = 1.5 radian. It is observed that there is good agreement between the results of the present problem obtained by FDM and the built-in numerical solver BVP4C technique.

The numerical solutions of the proposed model by FDM were obtained on the entire surface of the flow geometry. On the other hand, solutions by the built-in numerical solver BVP4C were obtained only at the leading edge of the surface of the proposed geometry for a similar form of the equations given in Equations (22)–(24) with the boundary conditions of Equation (25). Therefore, the results computed by the finite difference method were validated by the built-in numerical solver BVP4C at the leading edge. The comparison of the results by both methods supports the argument that main proposed method, which is FDM, applied for the current problem is accurate.

Table 1, Table 2 and Table 3 depict the numerical solutions for the skin friction, the rate of heat transfer, and the rate of mass transfer, respectively, computed by FDM and BVP4C at the leading edge for several values of the mixed convection parameter, *λ_t_*, at *X* = 1.5 radian when the rest of the dimensionless numbers are kept constant. From these tables, it is seen that increasing values of *λ_t_* lead to an enhancement of the skin friction, rate of heat transfer, and rate of mass transfer. 

Physically, it is endorsed that augmentation in *λ_t_* is basically due to an increase in the buoyancy force. The increase in buoyancy force causes an enhancement of the thermal expansion and temperature difference between the temperature of the surface of the sphere and the ambient temperature, due to which the temperature gradient increases and the slopes of the velocity and mass concentration increase. The percentage error at each value of *λ_t_* was calculated, and it is concluded that there are many favorable values of *λ_t_* where FDM and BVP4C are very close, which shows the agreement of the physical reasoning. Table 4, Table 5 and Table 6 display the behavior of the abovementioned quantities for different values of the thermophoretic coefficient, κ, at *X* = 1.5 radian when the remaining pertinent parameters are taken at their fixed values. From Table 4, Table 5 and Table 6, it is concluded that the skin friction and the rate of heat transfer increases, but the rate of mass transfer decreases. Again, the percentage error for each value of κ appears to be very small, t which shows the validation of FDM at the leading edge. 

## 5. Conclusions

In the current study, mixed convection heat transfer with thermophoretic transportation around the different positions of a sphere for various pertinent dimensionless parameters was performed numerically. The outcomes of the dimensionless parameters appeared in a flow model, such as the modified mixed convection parameter, thermophoresis parameter, Schmidt number, mixed convection parameter, thermophoretic coefficient, and Prandtl number, on the flow structure and heat transfer characteristics were highlighted. The results for the present governing model were shown in graphs and tables. Our findings are outlined below:

- From the graphical representation of the numerical results for the velocity profile, it can be seen that the velocity profile increases for increasing values of *λ_t_* and *λ_C_* but decreases when Pr and Sc are increased. Interestingly, it was noticed that the favorable position to obtain the maximum magnitude of the velocity profile is *X* = 1.5 radian.
- It can be observed from the findings about the temperature profile that this physical property is enhanced when Sc is augmented and reduced when Pr, *λ_t_*, and *λ_C_* are increased. The suitable circumferential point about the surface of a sphere to achieve the highest value is *X* = π radian.
- The results for the mass concentration illustrated in Figure 2, Figure 3, Figure 4 and Figure 5 show that the mass concentration rises for increasing values of Pr and declines as Sc, *λ_t_*, and *λ_C_* are enhanced. The station around a sphere, where the maximum value of the mass concentration was obtained, is *X* = π radian.
- It can be seen from Figure 6, Figure 7, Figure 8, Figure 9 and Figure 10 that there is augmentation in the skin friction owing to an increase in *N_t_*, *λ_t_*, and *λ_C_* and a reduction occurs when Pr and Sc are increased.
- The findings displayed in Figure 6, Figure 7, Figure 8, Figure 9 and Figure 10 indicate that the rate of heat transfer increases for increasing estimations of Pr, *λ_t_*, *λ_C_*, and *N_t_* and a decreasing attitude is viewed for the increasing values of Sc.
- The numerical results for the rate of mass transfer presented Figure 6, Figure 7, Figure 8, Figure 9 and Figure 10 depict that the rate of mass transfer rises for increasing values of Sc, *λ_t_*, and *λ_C_* and slows down when Pr and *N_t_* are augmented.
- It is shown in Figure 11a,b that the thermophoretic velocity at the surface of the proposed geometry is in the upper limit at position *X* = 1.5 radian, and in both cases, approaches to zero as the size of the particle is increased.
- In the end comparison of the numerical solutions of the skin friction, the rate of heat transfer and the rate of mass transfer for several values of *λ_t_* and κ, obtained by the finite difference method and MATLAB built-in numerical solver bvp4c, are shown in a tabular form. It was observed that the results of both techniques are in good agreement, which shows the validation of the numerical findings obtained by the proposed finite difference method applied to the primitive variable form for the current problem.

## Figures and Tables

**Table 1:** Numerical values of the skin friction obtained by the Finite Difference Method FDM and built-in numerical solver, boundary value problem of fourth order code BVP4C technique for different values of the mixed convection parameter, *λ_t_*, when *λ_C_* = 10.0, κ = 1.0, *N_t_*= 1.0, Pr = 0.71, and Sc = 1.0 at *X* = 1.5 radian.

$\lambda_{t}$	${\left(\frac{\partial U}{\partial Y}\right)}_{y=0}$		% Error
	FDM	BVP4C	
1.0	8.12161	8.12798	0.0784%
10.0	13.20016	13.20398	0.0289%
20.0	19.22151	19.22493	0.0178%
30.0	24.52932	24.53024	0.0037%
40.0	29.41793	29.41744	0.0017%
50.0	34.14011	34.140639	0.0015%

**Table 2:** Numerical values of the rate of heat transfer obtained by the FDM and built-in numerical solver BVP4C technique for different values of the mixed convection parameter, *λ_t_*, when *λ_C_* = 10.0, κ = 1.0, *N_t_* = 1.0, Pr = 0.71, and Sc = 1.0 at *X* = 1.5 radian.

$\lambda_{t}$	${\left(\frac{\partial \theta }{\partial Y}\right)}_{y=0}$		% Error
	FDM	BVP4C	
1.0	0.68709	0.68756	0.0684%
10.0	0.78215	0.78279	0.0182%
20.0	0.87263	0.87283	0.0229%
30.0	0.94489	0.94577	0.0930%
40.0	1.01645	1.01702	0.0560%
50.0	1.02183	1.02149	0.0332%

**Table 3:** Numerical values of the rate of mass transfer obtained by the FDM and built-in numerical solver BVP4C technique for different values of the mixed convection parameter *λ_t_* when *λ_C_* = 10.0, κ = 1.0, *N_t_* = 1.0, Pr = 0.71, and Sc = 1.0 at *X* = 1.5 radian.

$\lambda_{t}$	${\left(\frac{\partial \phi }{\partial Y}\right)}_{y=0}$		% Error
	FDM	BVP4C	
1.0	0.60516	0.60527	0.0181%
10.0	0.78805	0.78852	0.0596%
20.0	0.87532	0.87553	0.0239%
30.0	0.94610	0.94630	0.0211%
40.0	1.03342	1.03383	0.0397%
50.0	1.08060	1.08094	0.0314%

**Table 4:** Numerical values of the skin friction obtained by the FDM and built-in numerical solver BVP4C technique for different values of the thermophoretic coefficient, κ, when *λ_C_* = 10.0, *λ_t_* = 10.0, *N_t_* = 1.0, Pr = 0.71, and Sc = 1.0 at *X* = 1.5 radian.

k	${\left(\frac{\partial U}{\partial Y}\right)}_{y=0}$		% Error
	FDM	BVP4C	
0.1	12.15382	12.15397	0.0012%
1.0	13.31494	13.31459	0.0026%
2.0	15.02421	15.02438	0.0011%
3.0	16.37157	16.37155	0.0001%
4.0	17.08623	17.08643	0.0011%
5.0	17.73685	17.73644	0.0023%

**Table 5:** Numerical values of the rate of heat transfer obtained by the FDM and built-in numerical solver BVP4C technique for different values of the thermophoretic coefficient, κ, when *λ_C_* = 10.0, *λ_t_* = 10.0, *N_t_* = 1.0, Pr = 0.71, and Sc = 1.0 at *X* = 1.5 radian.

k	${\left(\frac{\partial \theta }{\partial Y}\right)}_{y=0}$		% Error
	FDM	BVP4C	
0.1	0.76252	0.76212	0.0524%
1.0	0.78554	0.78595	0.0521%
2.0	0.82730	0.82732	0.0024%
3.0	0.85465	0.85438	0.0316%
4.0	0.88616	0.88633	0.0001%
5.0	0.90459	0.90468	0.0099%

**Table 6:** Numerical values of the rate of mass transfer obtained by the FDM and built-in numerical solver BVP4C technique for different values of the thermophoretic coefficient, κ, when *λ_C_* = 10.0, *λ_t_* = 10.0, *N_t_* = 1.0, Pr = 0.71, and Sc = 1.0 at *X* = 1.5 radian.

k	${\left(\frac{\partial \phi }{\partial Y}\right)}_{y=0}$		% Error
	FDM	BVP4C	
0.1	1.95746	1.95710	0.0183%
1.0	0.77332	0.77343	0.0142%
2.0	0.42910	0.42906	0.0093%
3.0	0.41085	0.41086	0.0024%
4.0	0.43140	0.43108	0.0742%
5.0	0.44715	0.44719	0.0089%
